# Nature-inspired delivery of mitochondria-targeted angiotensin receptor blocker

**DOI:** 10.1093/pnasnexus/pgac147

**Published:** 2022-08-04

**Authors:** Jude M Phillip, Ran Lin, Andrew Cheetham, David Stern, Yukang Li, Yuzhu Wang, Han Wang, David Rini, Honggang Cui, Jeremy D Walston, Peter M Abadir

**Affiliations:** Department of Biomedical Engineering, Johns Hopkins University, Baltimore, MD, USA; Department of Chemical and Biomolecular Engineering, Johns Hopkins University, Baltimore, MD, USA; Institute for Nanobiotechnology, Johns Hopkins University, Baltimore, MD, USA; Department of Oncology, Sidney Kimmel Comprehensive Cancer Center, Johns Hopkins University School of Medicine, Baltimore, MD, USA; Department of Chemical and Biomolecular Engineering, Johns Hopkins University, Baltimore, MD, USA; Institute for Nanobiotechnology, Johns Hopkins University, Baltimore, MD, USA; Department of Chemical and Biomolecular Engineering, Johns Hopkins University, Baltimore, MD, USA; Department of Chemical and Biomolecular Engineering, Johns Hopkins University, Baltimore, MD, USA; Institute for Nanobiotechnology, Johns Hopkins University, Baltimore, MD, USA; Institute for Nanobiotechnology, Johns Hopkins University, Baltimore, MD, USA; Department of Chemical and Biomolecular Engineering, Johns Hopkins University, Baltimore, MD, USA; Institute for Nanobiotechnology, Johns Hopkins University, Baltimore, MD, USA; Department of Chemical and Biomolecular Engineering, Johns Hopkins University, Baltimore, MD, USA; Institute for Nanobiotechnology, Johns Hopkins University, Baltimore, MD, USA; Department of Art as Applied to Medicine, Johns Hopkins University School of Medicine, Baltimore, MD, USA; Department of Chemical and Biomolecular Engineering, Johns Hopkins University, Baltimore, MD, USA; Institute for Nanobiotechnology, Johns Hopkins University, Baltimore, MD, USA; Department of Medicine, Division of Geriatrics, Johns Hopkins University School of Medicine, Baltimore, MD, USA; Department of Medicine, Division of Geriatrics, Johns Hopkins University School of Medicine, Baltimore, MD, USA

**Keywords:** mitochondrial targeting, Losartan, angiotensin system, mitochondria

## Abstract

Mitochondria are critical regulators of cellular function and survival. We have previously demonstrated that functional angiotensin receptors embedded within the inner mitochondrial membrane modulate mitochondrial energy production and free radical generation. The expression of mitochondrial angiotensin II type-1 receptors increases during aging, with a complementary decrease in angiotensin II type-2 receptor density. To address this age-associated mitochondrial dysfunction, we have developed a mitochondria-targeted delivery system to effectively transport angiotensin type-1 receptor blocker—Losartan (mtLOS) into the inner mitochondrial membrane. We engineered mtLOS to become active within the mitochondria after cleavage by mitochondrial peptidases. Our data demonstrate effective and targeted delivery of mtLOS into the mitochondria, compared to a free Losartan, or Losartan conjugated to a scrambled mitochondrial target signal peptide, with significant shifts in mitochondrial membrane potential upon mtLOS treatment. Furthermore, engineered mitochondrial-targeting modalities could open new avenues to transport nonmitochondrial proteins into the mitochondria, such as other macromolecules and therapeutic agents.

Significance Statement We detail the development of a “nature-inspired” delivery system that directly targets angiotensin-II receptors within the mitochondria using a Mitochondrial Targeting Signal (MTS) peptide conjugated to Losartan. MTS allows the specific and selective recognition by the mitochondrial protein import machinery. Here, we also modified the mitochondria-targeted Losartan so that only the portion of the drug taken by the mitochondria becomes functionally activated after cleavage by mitochondrial peptidase. The development of this unique delivery allows us to selectively target the mitochondrial angiotensin receptors, which could also serve as a template for selective mitochondrial delivery of other drugs/peptides.

## Introduction

Mitochondria play a vital role in the regulation of energy metabolism, reactive oxygen species (ROS) production, and apoptosis. As a result, mitochondria provide an attractive drug target in the context of aging and disease. The primary cardioprotective benefits of angiotensin (Ang) receptor type-1 (AT_1_R) blockers (ARBs), such as Losartan (LOS), are believed to arise from systemic effects on blood pressure and vascular remodeling (1, 2). However, we and others have reported the existence of mitochondrial angiotensin receptors (mtAT_1_R and mtAT_2_R) (3, 4). These findings have enhanced our understanding of the renin–angiotensin system (RAS) beyond blood pressure regulation to a broad intra- and extracellular system, which is integrated from the cell surface to nuclei and mitochondria (4–6). Increased mitochondrial AT_1_R has been linked to the mitochondrial free radical generation and oxidative stress (3, 7). While systemic administration of ARBs has been documented to improve mitochondrial functions, such administration is limited and often associated with toxicities such as renal function impairments in older adults. Therefore, there is a need to develop a selective mitochondria-targeting AT_1_R blocker for the treatment of age-associated mitochondrial decline.

Delivering a drug to the mitochondria is not trivial. Mitochondria are unique subcellular organelles containing DNA and RNA, which are translated locally within the mitochondria and the nucleus. These externally derived proteins and molecules are imported into the mitochondria via a system of outer- and inner-membrane-bound protein complexes (8, 9), which mediate delivery to the appropriate mitochondrial compartment. This import process is regulated by an N-terminal presequence that is located on the protein itself—these “tags” are known as Mitochondrial Targeting Signal peptides (MTS). This MTS-tag serves as an entry barcode, indicating where the protein should be delivered. Once the protein is present in the desired mitochondrial compartment, the MTS portion of the protein is cleaved by mitochondrial peptidases, allowing the protein to fold into an active functional state. Due to the high specificity of MTS peptides, they could enable the effectual transport of nonmitochondrial proteins and bioactive agents directly into the mitochondria, as a proverbial “Trojan horse.” Here, we report the design and development of a functional drug delivery system using MTS peptides covalently linked to Losartan for direct delivery into the mitochondria.

## Results

### Strategy to deliver bioactive agents into the mitochondria

**Fig. 1.**
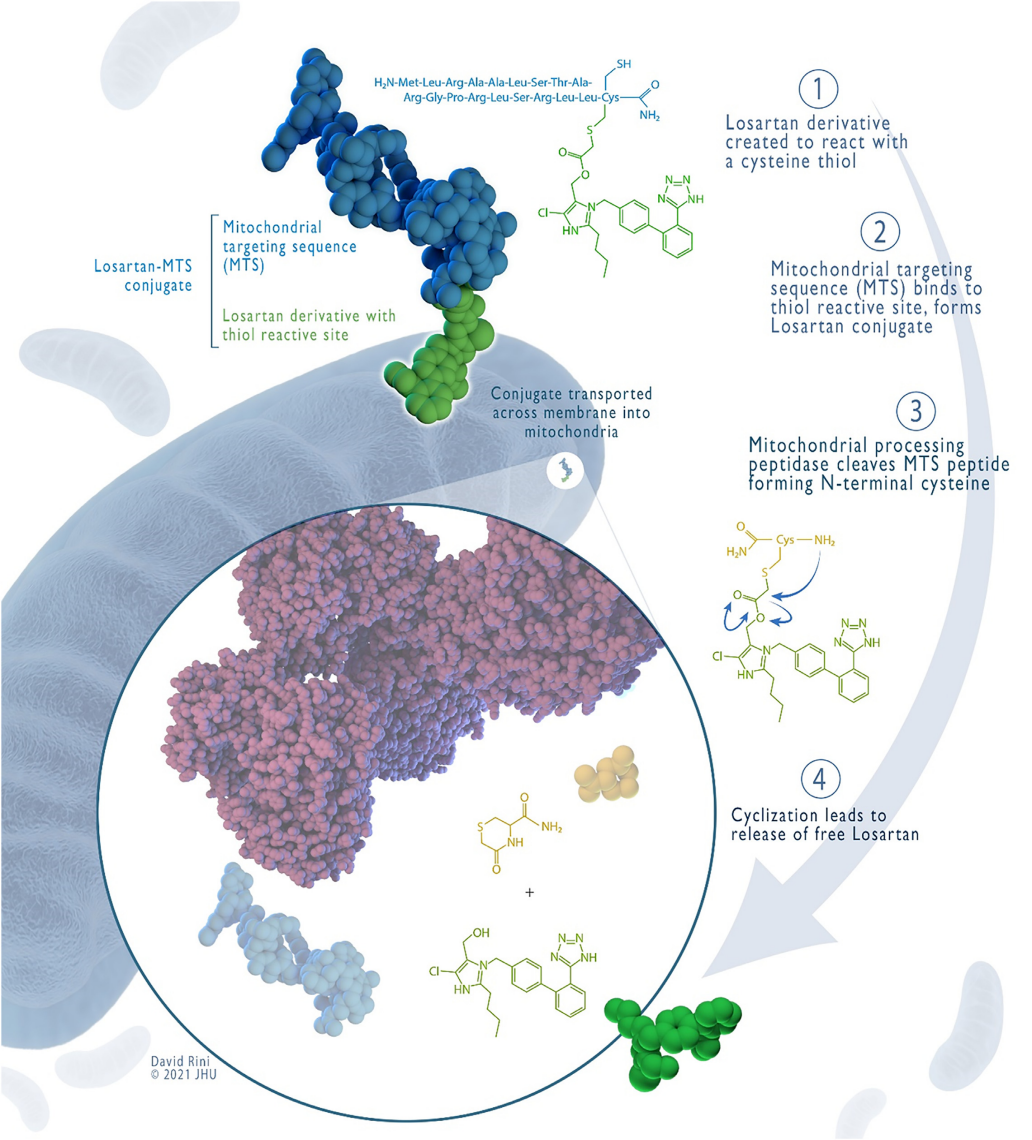
(A) Schematic representation of the biological process of targeting a protein to the mitochondria using mitochondrial targeting sequence (MTS) and how the nature-inspired mitochondria-targeted Losartan (mtLOS) was developed. (B) Sequences of the three different MTS and scrambled MTS peptides that were used for this study.

**Fig. 2.**
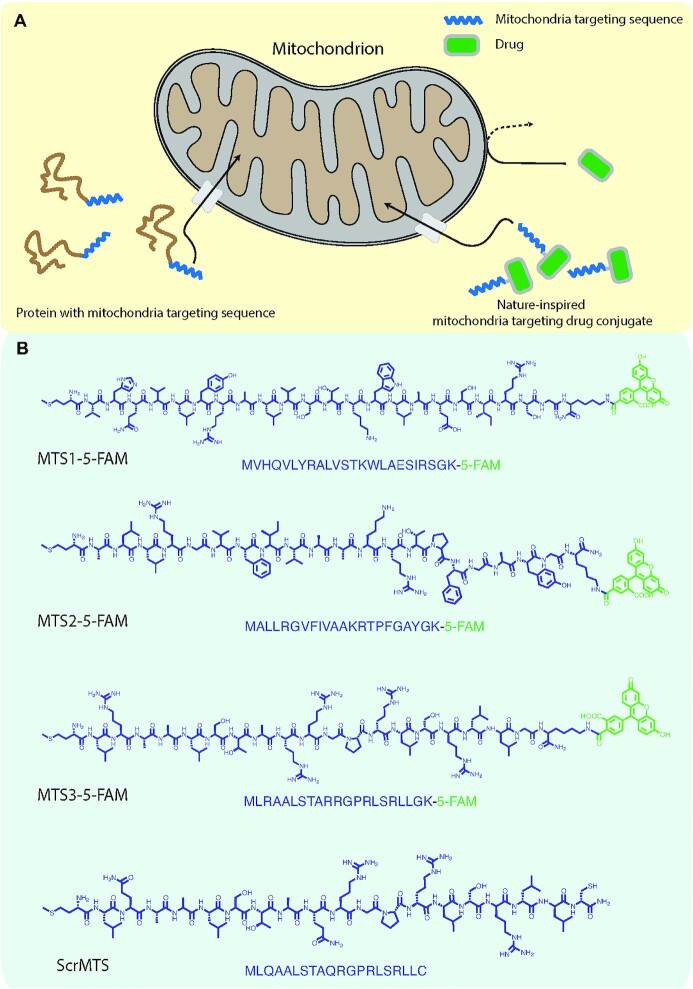
Schematic illustration of the design, synthesis, and mechanism of uptake of the mitochondrially targeted Losartan. Upper left: 3D model and chemical structure of the Losartan–MTS conjugate, MTS peptide (blue), and Losartan derivative (green). Lower left: a zoom-in the schematic of the cleavage of the Losartan–MTS conjugate and the release of free Losartan. Upper and lower right: synthesis and mechanism of uptake and cleavage of the Losartan–MTS conjugate, step1: the synthesis of a Losartan derivative that can react with a cysteine thiol, step 2: the synthesis and purification of a mitochondrial targeting sequence peptides and the subsequent conjugation of the modified Losartan to the mitochondrial targeting sequence. This allows specific and selective uptake by the mitochondrial protein import machinery. Step 3: once inside the mitochondria, the cleavage of the MTS peptide by mitochondrial processing peptidases results in the release of free Losartan.

To directly target AT_1_R within the mitochondria, we have designed a mitochondria-targeted Losartan conjugate, mtLOS. Our process included (1) the selection, synthesis, and purification of MTS peptides (10, 11), (2) the synthesis of a Losartan derivative that can react with a cysteine thiol (to allow for the conjugation to macromolecules), and (3) the subsequent conjugation of these two components. This allows specific and selective recognition by the mitochondrial protein import machinery (9, 11–14). Once inside the mitochondria, the cleavage of the MTS peptide by mitochondrial processing peptidases results in the release of free Losartan (Fig. 1A). To visualize and quantify the MTS-mediated selective delivery of mtLOS to the mitochondria, we also incorporated a fluorescent tag (5FAM) into the MTS peptides (see the synthesis of MTS–Losartan–5-FAM in “Materials and Methods”). Figure 2 summarizes the synthesis and the mechanism of uptake of the Losartan–MTS conjugate.

### Mitochondrial colocalization of mtLOS

**Fig. 3.**
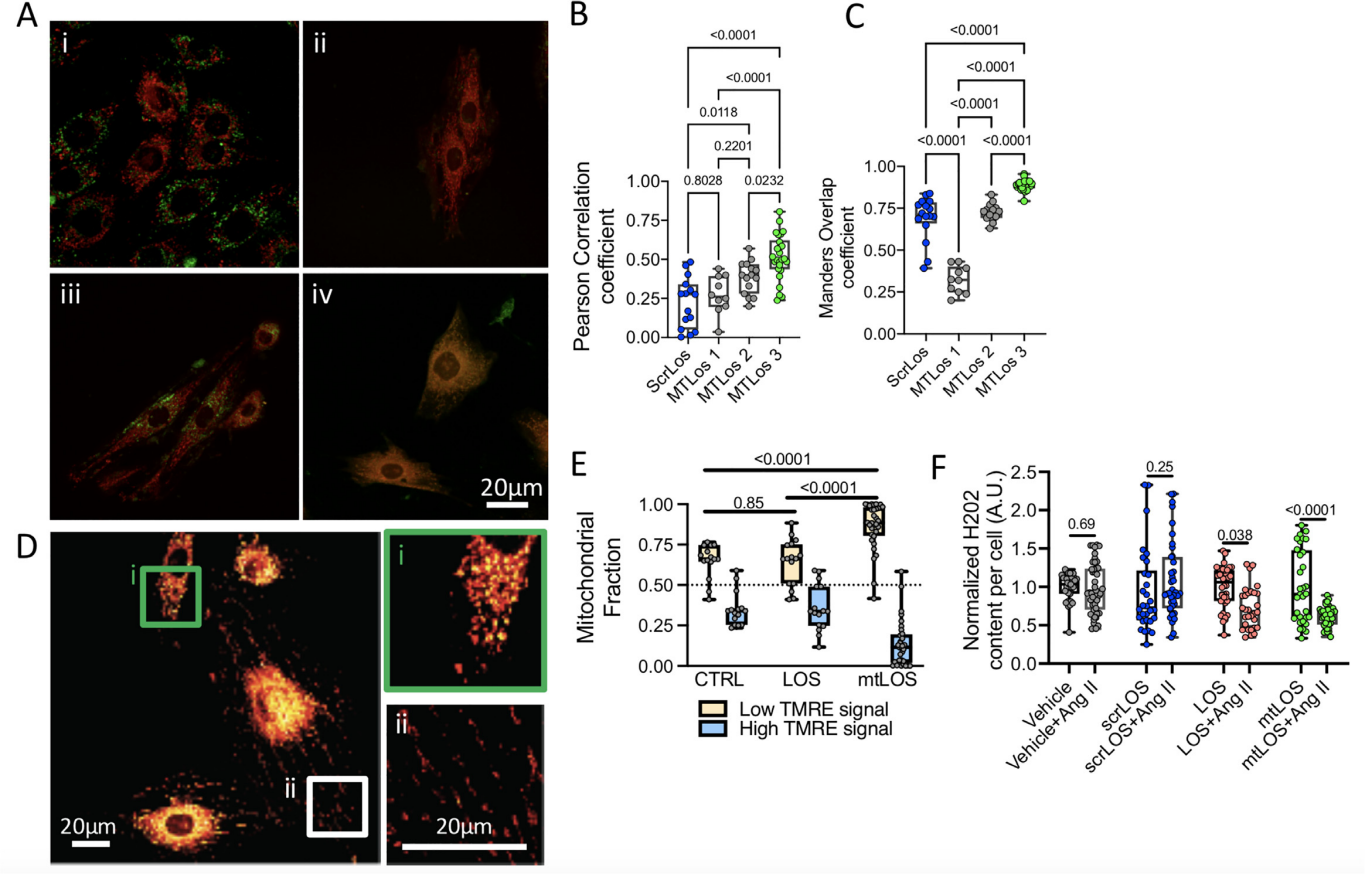
Mitochondrial targeting and functional consequences. (A) Representative confocal images showing colocalization of mitochondrial networks (red—TOM20) with different mitochondrial targeting sequences (green): (i) scrLOS3, (ii) mtLOS1, (iii) mtLOS2, and (iv) mtLOS3. (B) and (C). Colocalization quantification is based on the Pearson’s correlation coefficient (B) and Mander’s overlap coefficient (C). Number of cells analyzed per conditions were: scrLOS3 (*n* = 18), mtLOS1 (*n* = 12), mtLOS2 (*n* = 17), and mtLOS3 (*n* = 26). (D) Representative images of TMRE-stained mitochondrial networks, insets show sparse (i) and dense (ii) mitochondrial networks. (E) Quantification of a mitochondrial fraction having high versus low membrane potential per cell upon treatment with scrLOS (*n* = 15), free Losartan (*n* = 17), and mtLOS3 (*n* = 32). (F) Quantification of superoxide content per cell, vehicle (*n* = 35), vehicle + Ang II (*n* = 48), scrLOS (*n* = 30), scrLOS + Ang II (*n* = 39), free Losartan (*n* = 40), LOS + Ang II (*n* = 30), mtLOS (*n* = 32), and mtLOS + Ang II (*n* = 37).

Given that different MTS peptides have different efficacy and specificity in targeting mitochondria, we formulated three MTS sequences and a scrambled control sequence. Each molecule was conjugated with 5FAM, providing the ability to assess the efficacy of mtLOS delivery into the mitochondria (Fig. 1B). To identify which MTS displayed the most effective delivery into the mitochondria, cells were seeded at low density and allowed to spread on collagen-1 coated glass surfaces (50 µg/ml). Cells were briefly permeabilized with 8 nM digitonin for 15 minutes and then incubated with each of the four molecules for 12 hours (Fig. 1B). Samples were washed with PBS and then fixed with 4% paraformaldehyde before staining for the mitochondrial membrane using TOM20 (Santa Cruz). After staining, samples were imaged using a Nikon A1 confocal microscope. Effective delivery into the mitochondria was quantified based on the spatial colocalization of 5-FAM and the outer mitochondrial membrane (TOM20), serving as the proxy for Losartan delivery (Fig. 3A). Z-stacks of individual cells were acquired, and the efficacy of delivery within the mitochondrial network was quantified based on the Pearson’s correlation coefficient and Mander’s overlap coefficient (Fig. 3B and C). Results indicated a significant increase of mtLOS into the mitochondria for mtLOS3, with a Pearson’s correlation and Mander’s overlap coefficient of 0.59 and 0.88, respectively, compared to 0.17 and 0.70 for the scramble control sequence (scrLOS). mtLOS3 was, therefore, selected for functional assays and compared to scrLOS.

### Quantification of mitochondria function upon exposure to mtLOS3 and scrLOS

To elucidate downstream functional effects of Losartan delivery into the mitochondria, we assessed mitochondrial responses based on changes in the membrane potential upon exposure of cells to either mtLOS3 or scrLOS. Cells were briefly permeabilized, then incubated with mtLOS3 or scrLOS for 12 hours, after which cells were washed and incubated with mitochondrial membrane-potential marker TMRE (ThermoFisher Scientific). After TMRE incubation, cells were washed thoroughly with PBS, resuspended in fresh cell culture media, and imaged using a Nikon A1 confocal microscope equipped with a stage-top environment control unit (Tokai Hit). Individual cells were imaged, and z-stacks were acquired for each cell per condition. After background correction to optimize signal-to-noise, the maximum intensity projection was used to compute the membrane potential per cell as the sum mitochondrial intensity per cell (Fig. 3D). Analysis revealed decreases in the number of mitochondria per cell (number of TMRE-positive pixels) and the total mitochondrial membrane potential per cell (intensity per positive pixel), with no difference in the mean mitochondrial potential (average TMRE-positive pixel intensity) per cell.

Upon visual inspection of the mitochondrial network, we observed an apparent dichotomy in the fraction of high- and low-intensity mitochondria per cell after mtLOS3 and scrLOS treatment. To quantify this, we computed the intensities of all mitochondria-positive regions per cell (per-pixel) and combined the distribution of intensities for all cells in both treatment conditions. Using the median of the distribution, we split the intensity profiles into high versus low intensities, then computed the fraction of mitochondria classified as high or low per cell for each condition. This analysis revealed a polarization of mitochondria, with cells treated with mtLOS3 having a higher fraction of low-intensity mitochondria (∼86%) compared to scrLOS-treated cells (∼65%; Fig. 3E). A similar trend was also observed in cells treated with free Losartan with a 70% fraction of low-intensity mitochondria.

This significant polarization of mtLOS3-treated cells having higher fractions of mitochondria with low membrane potential prompted us to assess the production of superoxide (O_2_) content per cell as a function of mtAT_1_R. We stained live cells exposed to either scrLOS, free Losartan, or mtLOS3 and quantified the superoxide content per cell. Image-based quantification showed no significant differences in the superoxide content per cell after treatment with scrLOS, free Losartan (5 uM), or mtLOS3. However, upon cotreatment with 10 nM ANG II—blocking AT1R and stimulating AT2R, cells treated with mtLOS3 exhibited significantly lower superoxide content per cell, with a similar trend observed with free Losartan (Fig. 3F). Furthermore, quantifying nitric oxide (NO) and ROS in either condition showed no statistically significant differences. Taken together, our data demonstrate success in selecting a mitochondrial targeting sequence (MTS) that, when bound to Losartan, demonstrates an active engagement with its mitochondrial AT_1_R target.

## Discussion

The identification of a functional intramitochondrial angiotensin system provided new insights into the interface between RAS and mitochondria. A critical hurdle in studying the mitochondrial angiotensin system is distinguishing the effects of AT1R directly at the mitochondria from peripheral effects, which indirectly lead to mitochondrial changes. To overcome this obstacle, we have developed a specific delivery system that targets mitochondria with Losartan via conjugation of Losartan to an MTS peptide. Our data show that MTSs can be conjugated to macromolecules, with effective delivery of mtLOS3 into the mitochondria, as compared to the scrLOS. In addition, we show that both mtLOS3 and free Losartan increased the fraction of mitochondria with a lower level of mitochondrial potential, with cotreatment of cells with either free Losartan or mtLOS3 and Ang-II leading to a reduction in mitochondrial hydrogen peroxide (O_2_). However, a more robust effect was observed for cotreatment with mtLOS relative to cotreatments with free Losartan.

The mechanism of how different MTS direct the import of precursor proteins into mitochondria remains largely obscure. Of the three MTSs, MTS3 showed the highest efficacy in delivering the conjugated Losartan in cardiomyocytes. MTS3 is a rat liver aldehyde dehydrogenase (10). However, it remains unclear why MTS3 had the highest efficacy in delivering Losartan to the mitochondria. A number of reasons could contribute to this specificity, including experiment conditions, cell type, Losartan chemical, and physical properties. Treatment of cardiomyocytes with mtLOS3 led to a significant increase in the number of mitochondria with low activity.

mtLOS was modified in a way that only the portion of mtLOS taken up by the mitochondria becomes functionally activated upon cleavage by mitochondrial peptidase. We observed that the use of mtLOS3 when cells were cotreated with Ang II led to a reduction in the Ang II-driven increase in O_2_. This function is analogous to systemically administered Losartan. The failure of scrambled Losartan to protect against the Ang II-driven increase in H_2_O_2_ argues against a nonmitochondrial action of Losartan.

MTS allows for specific and selective recognition by the mitochondrial protein import machinery. Though further work is needed to allow for systemic administration of mtLOS to live animals, this new technique will open the door for an array of experiments aiming at selectively targeting the mitochondrial ANG system while avoiding systemic side effects. Given that systemic use of current ARBs is limited in many older individuals by low blood pressure and renal impairment, the further development of this technology may allow systemic administration of targeted ARBs that bypass the PAS, avoiding the effects on kidney and blood pressure.

## Materials and methods

### Synthesis of MTS–Losartan–5-FAM conjugate

In the synthesis of chloroacetyl Losartan ester, Losartan (50 mg, 118 µmol), chloroacetic acid (13.4 mg, 142 µmol), and dimethyl-4-aminopyridine (2.8 mg, 27 µmol) were dissolved in 4 ml Tetrahydrofuran (THF). After cooling the solution to 0°C, diisopropylcarbodiimide (23 µl, 142 µmol) was added and continuously stirred for 90 minutes at 0°C. The mixture was allowed to warm to room temperature. The reaction was monitored by thin-layer chromatography. After overnight stirring, the reaction was deemed complete. The mixture was then diluted with 10 ml dichloromethane (DCM) and washed with 0.1 M 15 ml HCl and 15 ml brine. The organic phase was dried over Na_2_SO_4_, and solvents were removed in vacuo. Further drying under vacuum gave chloroacetyl Losartan as a white solid that was shown by NMR to be > 90% pure (69 mg, quantitative yield).

The Fmoc-protected peptide Fmoc-MLRAALSTARRGPRLSRLLCK-(Mtt) was synthesized on a Rink MBHA resin (250 µmol) using a Focus XC automated peptide synthesizer (AAPPTEC, Louisville, KY, USA) and standard Fmoc solid-phase synthesis protocols, using 20% 4-methylpiperidine in dimethylformamide (DMF) for Fmoc deprotection, amino acid:HBTU:DIEA = 4:3.98:6 (relative to the amino resin) in DMF for coupling. To conjugate 5-FAM, the Mtt protecting group was deprotected using 10 ml TFA/TIS/DCM (4:5:91), shaking for 5 minutes intervals until no yellow color developed on the addition of the reagent. The resin was then shaken with a solution of DIEA in DMF to neutralize residual TFA. 5-FAM (142 mg, 377 µmol), HATU (140 mg, 368 µmol), and DIEA (95 µl, 563 µmol) were dissolved in 5 ml DMF. The solution was then added to the resin and shaken for 6 hours. After washing (3 × DMF, 3 × DCM), a positive Kaiser test for free amine was obtained, so the coupling step was repeated. The N-terminal Fmoc group was removed, and the peptide was cleaved from the resin using 10 ml TFA/TIS/H2O/EDT (92.5:2.5:2.5:2.5) for 3 hours. The peptide was isolated by concentration in vacuo followed by precipitation in cold Et_2_O. The crude product was purified by reverse phase HPLC and analyzed by MALDI-TOF mass spectrometry.

To synthesize MTS–Losartan–5-FAM conjugate, MTS3–5-FAM (10.6 mg, 3.9 µmol) and chloroacetyl Losartan (2.9 mg, 5.8 µmol) were first dissolved in 500 µl DMF, and then DIEA (1.65 µl, 9.8 µmol) was added. The mixture was allowed to react at 4°C for 3 days, after which HPLC analysis showed 60% conversion to the desired product. The mixture was diluted to 8 ml with 0.1% aqueous TFA and purified by reverse phase HPLC. Product-containing fractions were combined and lyophilized to give MTS–Losartan–5-FAM as a yellow powder.

### Quantification of mitochondrial potential and superoxide content

For all experiments, rat cardiac cells (H9C2) were plated at low density onto cell culture treated glass substrates (Griener) in Dulbecco’s Modified Eagle Medium (Gibco) supplemented with 10% FBS (Hyclone) and 1% penicillin/streptomycin (Gibco). For quantifying mitochondria, potential cells were incubated with TMRE mitochondrial membrane potential kit (ThermoFisher) for 30 minutes, and then the supernatant was removed and gently washed with PBS. Z-stacks were acquired on a Nikon A1 confocal microscope, with experiments performed in biological duplicates or triplicates. Images were processed using a combination of ImageJ and Matlab. Briefly, a mask was generated for each cell, delineating its boundaries so that we can have single cell quantifications. We performed a background correction, then the maximum projection of the z-stacks for each cell was used to generate the spatial intensity profile per cell, from which the mitochondrial network footprint, summation, and average intensity per cell were computed. These parameters were calculated on a pixel-basis. To compute the fractional abundance of high and low signal mitochondria, we generated pixel values of the mitochondrial networks for each cell and compiled them into a single vector. Then from this distribution of values, we determined the median intensity, which was used to delineate high (above median) and low (below median) intensity profiles. For each cell per condition, we then computed the fraction of high-intensity and low-intensity pixels.

To compute the superoxide (O_2_) content per condition, we utilized Mitosox kit (ThermoFisher). Cells were incubated with kit reagents per the manufacturer’s protocol, and cells were imaged using a Nikon A1 confocal microscope. Z-stacks for individual cells were acquired, and the signal was computed per condition based on similar approaches described above.

### Statistical analysis

Results for all experiments performed were conducted in biological duplicate or triplicate. Statistical significance was determined based on either t test or one-way ANOVA. In all plots shown, the *P*-values are indicated.

## Data Availability

All data is included in the manuscript.
